# Analysis of food policymaking through a food systems lens: a review of analytical frameworks

**DOI:** 10.1017/S1368980025100906

**Published:** 2025-08-12

**Authors:** Isobel Stanley, Celine Murrin

**Affiliations:** School of Public Health, Physiotherapy and Sports Science, University College Dublin, Belfield, Dublin 4, D04 V1W8, Ireland

**Keywords:** Food policy, Food systems, Sustainability, Policymaking processes

## Abstract

**Objective:**

This paper aims to summarise the frameworks currently used to analyse food policymaking processes and to critically assess whether those frameworks can be applied to the analysis of integrated, ‘systems’ approaches to policymaking.

**Design:**

Two electronic databases were searched to identify publications analysing food policymaking processes. Data from the publications were charted using an iterative coding process, and details of the underlying analytical frameworks were recorded. Identified frameworks were evaluated using theories of systems approaches to food policy development.

**Setting:**

Governmental food policy at the supranational, national and local levels.

**Results:**

The search process yielded 532 results. After screening, a final forty-three publications and twenty-four frameworks were identified. In the studies, frameworks were used to analyse agenda-setting, stakeholder networks, policy coherence and development of national food and nutrition policies. All twenty-four frameworks allowed for analysis of actors and context in policymaking processes, while space for considering policy coherence featured less (*n* 11).

**Conclusions:**

Three frameworks were highlighted as particularly applicable to the context of food systems approaches to policymaking. The application of analytical frameworks for policymaking processes is limited in food policy research. However, this review demonstrates that there are considerable benefits to using such frameworks to understand the ideas, knowledge, power and decision-making that lead to food policy development. This is particularly useful in understanding the complex stakeholder networks and policy coherence necessary for successful policies for sustainable food systems.

Food systems drive and are driven by today’s major public health challenges. They operate at a high cost to human and environmental health. For example, inequalities within systems drive food insecurity, with 2·8 billion people unable to afford a healthy diet in 2022^(1)^. In turn, sub-optimal diets are a major risk factor for disease and death^(2)^. Food systems also contribute to and are vulnerable to environmental challenges such as greenhouse gas emissions, biodiversity loss and resource overuse^(3,4)^. As such, there is a pressing need for a sustainable system ‘*that delivers food security and nutrition for all, in such a way that the economic, social and environmental bases to generate food security and nutrition for future generations are not compromised*’^(5)^.

Existing policies and policymaking processes fail to address these systemic issues in the food system. Food policy, ‘*any government action or decision concerning the production and processing of food, its impact on public health and wellbeing, the environment and natural resources*’^(6)^, is often developed in siloes across government departments. Policy agendas for health, agriculture, trade and environment are frequently made in isolation. Furthermore, food systems have historically been oriented towards maximising profits for businesses and enhancing national trade competitiveness, reinforced by policies that prioritise high-volume and efficient production^(7–9)^.

This siloed food policy approach has led to disconnects and incoherence between policies targeting different parts of the food system and has limited the opportunities for addressing the interconnected challenges across the system^(10)^. To transition to a sustainable food system, coherent and integrated food policies that consider the interconnections within the system between health, livelihoods and environment are fundamental. The Organisation for Economic Co-operation and Development (OECD), in their report ‘Making Better Policies for Food Systems’, emphasises the need for a holistic systems approach to policymaking to address the interconnected ‘triple challenges’ of food security, livelihoods and environmental sustainability^(11)^. The report recommends aligning policies across these domains to account for benefits and trade-offs. For example, a policy reducing subsidies for dairy and meat production might result in lower greenhouse gas emissions and population red meat consumption, providing co-benefits for the environment and health. A possible trade-off, however, is a negative impact on employment in the meat and dairy sectors and on farmer livelihoods^(12)^. Another key recommendation is the adoption of inclusive, multi-stakeholder approaches in policy processes to address conflicts in facts, interests and values. The report also advocates for coordinated, cross-sectoral policymaking to enhance policy coherence, ensuring that objectives in one sector do not undermine those in another.

Affecting positive change towards these holistic approaches requires a clear understanding of how and why food policies are currently made. To achieve this, we also need to understand how existing theoretical approaches to analysing public policymaking processes can and have been applied to food-related domains and critically assess how conducive existing paradigms are to the incorporation of a food systems approach.

Theories and frameworks for the analysis of policy processes have long been used in political science research^(13)^. These frameworks are used to identify policy decisions and why they have been made. They analyse elements of policymaking such as policy actors (the organisations or individuals who take action), networks and subsystems (actor relationships, influence and power dynamics), policy framing (how issues are portrayed based on ideas and actor beliefs) and policy context (broader environment including socio-economic conditions, government infrastructure, existing policies and catalysing events)^(14)^. This paper aims to summarise the frameworks used in the literature to analyse and evaluate public policymaking processes in the food policy domain.

Using political science theories and frameworks to understand food policymaking contributes to a greater understanding of how and why policies are made and provides insight into the different conditions that are required for a policy to succeed across contexts. Due to the cross-cutting nature of food policies and the need for a holistic approach to their development, analyses of food policymaking processes require a combination of concepts that might differ from those appropriate to other policy domains. Analyses should consider the policy context, actor interactions and framing of policy problems and policy coherence and integration (the joining-up of policy processes to align goals and policies across domains and government levels). To this end, this paper also aims to assess how applicable the identified frameworks are to the analysis of integrated food systems approaches to policymaking.

Following increased recognition of the importance of political and structural determinants of health^(15)^, there have been increased calls in public health and political science domains for research that provides a deeper understanding of political processes behind the development of public policies that impact health^(13,16)^. To date, in public health and nutrition research, policy analysis has focused largely on policy evaluation and impact, policy content and the translation of science into policy, with few studies using political science theories^(13,16–18)^. While these types of analyses contribute to the understanding of public policymaking processes, they lack an understanding of the political processes integral to policymaking. A 2016 review of political science theories in public health nutrition research^(18)^ found an increase in the use of policy process frameworks such as the Advocacy Coalition Framework (ACF)^(19)^ and Kingdon’s multiple streams theory^(20)^ over time. However, the authors ultimately concluded that political science theories were underutilised in nutrition policy research. Similar reviews were conducted in the context of obesity prevention policies^(17)^ and health promotion research^(16)^. These reviews also concluded that the use of political science theories was limited. While Clarke *et al*. provided a summary of the analytical frameworks used, the three reviews^(16–18)^ focused primarily on the frequency of use of political science theories and frameworks in research and how that has changed over time. To date, reviews of political science methodologies in public health and nutrition have not critically assessed their use in analysing multisectoral food policies or their applicability in the context of integrated food systems approaches to policymaking.

By providing an overview of the analytical frameworks used to evaluate public food policymaking processes and critically assessing whether they can be applied to the analysis of integrated, food systems approaches to policymaking this paper aims to (1) provide an understanding of the existing use of political science frameworks and theories in food policy research and (2) to evaluate these analytical frameworks and their applicability in the context of integrated food systems approaches to food policymaking.

## Methods

The review was completed in two stages: (1) a systematic search was conducted (following the PRISMA guidelines^(21)^) to identify analytical frameworks used in peer-reviewed literature to evaluate public food policymaking processes; (2) the analytical frameworks identified were evaluated in terms of their applicability to integrated food systems approaches to policymaking using criteria based on OECD recommendations for ‘making better food policies for food systems’^(11)^.

### Data sources and search strategy

Two electronic databases were systematically searched to identify relevant literature: Web of Science Core Collection and Proquest (International Bibliography of the Social Sciences, Politics Collection, Social Science Database and Sociology Collection). Study titles, abstracts and keywords were searched for the following keywords: (‘policymaking’ or ‘policy develop*’ or ‘policy process*’ or ‘policy formulation’) and (‘analysis’ or ‘discourse’ or ‘appraisal’ or ‘assess’ or ‘evaluate’) and (‘food polic*’ or ‘nutrition polic*’ or ‘agriculture polic*’).

### Study selection

Inclusion and exclusion criteria were developed by both authors. Only peer-reviewed articles written in the English language were considered for inclusion. An initial screening of titles and abstracts was followed by a full-text review conducted by the first author. Studies were included or excluded based on the criteria in Table 1.

**Table 1.** Inclusion and exclusion criteria for studies containing analytical frameworks for policymaking processes

Inclusion criteria
The article is in a peer-reviewed journal indexed in Web of Science or Proquest.
The article is in English.
The article reports on analyses of food policymaking at the supranational, national, state and local government levels.
Analyses targeted at the first three steps in the public policy cycle^(22)^: (1) agenda-setting, (2) policy formulation and (3) decision-making or the policy change process.
Analyses based on an analytical framework.
Exclusion criteria
Articles relating to non-governmental policies, for example, organisation level policies.
Articles analysing policy evaluation or impact only.
Opinion pieces, commentaries, books, book chapters and theses.

The public policymaking process is often organised using the policy cycle^(22)^. Though not always sequential, the process is divided into the following stages: agenda-setting, formulation, implementation, evaluation and policy maintenance, succession or termination. Our research centres on agenda-setting (identifying and defining the policy problem and deciding what deserves government attention) and policy formulation (setting policy objectives and selecting policy instruments) as the activities undertaken during these stages are critical to implementing a food systems approach. As such, studies were excluded if the analysis related to non-governmental or organisational-level policies or was focused on policy evaluation. Grey literature, opinion pieces, commentaries and books were also excluded. Finally, as the second aim of the paper was to evaluate analytical frameworks used in food policy research, where the use of an explicit framework was not apparent in the full-text review, the study was excluded.

#### Data charting

The aim of the data charting was to identify the analytical frameworks used in the selected review papers. The article content was reviewed using an iterative coding process. This included the study setting, research question, data collection method, framework of analysis, topic and policymaking stages addressed. Data were inputted into Microsoft® Excel for analysis.

#### Selection and analysis of frameworks

To answer our second research question, the frameworks of analysis used in the forty-three review papers were extracted and rated independently on how applicable they are for the analysis of integrated food systems approaches to policymaking.

An initial categorisation and screening of the frameworks led to the selection of twenty-four unique frameworks 3. The frameworks were categorised based on their primary focus in the policy cycle^(22)^: ‘all policy cycle stages’; ‘agenda-setting’; ‘agenda-setting and policy formulation’; and ‘policy formulation and implementation’. Two review papers used a policy cycle heuristic as a framework of analysis; these are not generally considered analytical tools^(22)^ and were excluded from this second-stage analysis. Additionally, as some frameworks were used in more than one study, duplicate frameworks were removed.

The remaining frameworks (*n* 24) were rated on their application to analyse food systems approaches using three criteria described below.

As described previously, the OECD report ‘Making Better Policies for Food Systems’^(11)^ sets out recommendations for holistic food policymaking to address key food system challenges. These recommendations concern inclusive multi-stakeholder approaches, coordinated cross-sectoral policymaking and policy coherence. Further to this, Parsons *et al*. propose that irrationalities and disconnects in the food system can be effectively addressed through policy integration horizontally across governments, vertically between government levels or between inside and outside government actors^(23)^. In understanding how the recommendations above are operationalised, we also need to consider the context or environment in which the policy is developed and how the policy problem is framed by different actors and across different sectors^(14)^. Based on these recommendations and definitions, the following three criteria were developed for evaluating the analytical framework: (1) analysis of policy context and framing, (2) analysis of multi-stakeholder involvement and (3) analysis of policy coherence and integration processes. The criteria help to ensure that the frameworks can be applied to understand and evaluate food policymaking processes in the context of integrated systems approaches. The frameworks were rated as full, partial or none under each criterion, based on how comprehensively they included each criterion (Table 2).

**Table 2.** Benchmark definitions for rating frameworks on their capacity to support integrated food systems policy analysis

Criterion	Full	Partial	None
Analysis of policy context and framing	The framework enables a comprehensive assessment of policy framing from a systems perspective and the influence of ideas and issue characteristics on agenda-setting and policy goals. It supports the analysis of whether policy problems are recognised as cross-cutting and whether whole-of-government responses are considered. It also allows for the analysis of the political, economic, social and cultural factors shaping policy development and processes.	The framework addresses some contextual or framing dimensions but lacks a systems perspective or does not fully consider cross-sectoral analysis of policy context factors.	The framework does not support assessment of policy context, framing or systemic drivers of policy development.
Analysis of multi-stakeholder involvement	The framework enables a comprehensive assessment of multi-stakeholder engagement. It considers the range of actors and the completeness and quality of engagement processes and examines power dynamics and stakeholder interactions.	The framework allows for the identification of stakeholders but only partially assesses power dynamics, actor values or mechanisms for stakeholder engagement.	The framework does not substantially address stakeholder involvement or actor dynamics.
Analysis of policy coherence and integration processes	The framework enables a comprehensive assessment of policy coherence and integration across sectors and governance levels. It allows for the analysis of horizontal and vertical alignment of policy goals and objectives and considers measures of identifying trade-offs and spillovers.	The framework addresses some aspects of coherence or integration but does not provide a full analysis across both horizontal and vertical dimensions or of trade-offs and synergies.	The framework does not address policy coherence, integration or interactions between policy goals and objectives and governance levels.

For instance, under ‘analysis of multi-stakeholder involvement’, frameworks that were rated ‘full’ prompted analysis of actor values and beliefs, power and the nature of their engagement in the policy process. For example, the ‘Power Cube Framework’^(24)^ was rated ‘full’ as it prompted analysis of actor beliefs, power and the nature of their engagement in the policy process. Those that were rated ‘partial’ under the same criterion prompted a higher-level analysis, for example, listing the range of actors involved. In contrast, Shiffman’s ‘Agenda-setting Framework’^(25)^ was rated ‘partial’ as it includes prompts to list the range of actors involved in the policy process but was less prescriptive in its analysis of their role and how they shape the political process. A full table of rating justifications for the frameworks is included in the supplementary material.

## Results

**Figure 1:**
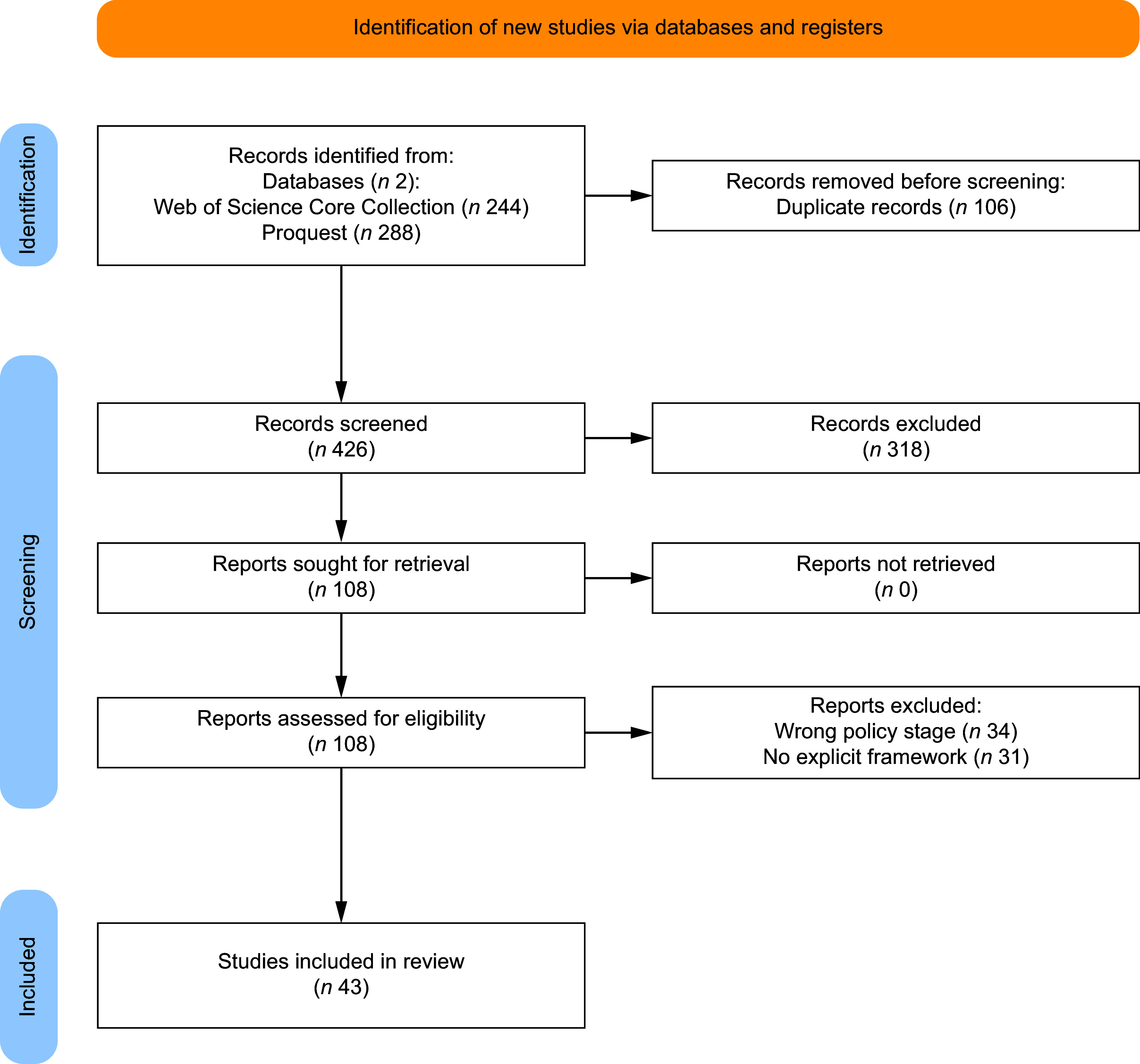
PRISMA flowchart of literature search and selection of the inclusion process^(21]^

The database search produced 532 results (Fig. 1). After the removal of duplicates and the title and abstract screening, 108 studies remained to be reviewed. During the full-text screening, thirty-four papers were excluded as they focused on policy impact and evaluation, and thirty-one papers were excluded for not explicitly articulating an analytical framework. At the end of the screening process, forty-three studies were included in the final review.

### Data charting

Of the forty-three studies included (Table 3), the majority of studies focused on analysing the policy context and on agenda-setting analysis (*n* 25). The remaining studies conducted analyses of stakeholder networks and participatory processes (*n* 5), policy integration, coherence and coordination in policymaking processes (*n* 9) and the development of local and national food and nutrition policies (*n* 3). All studies used qualitative methods in their analyses, including either case studies (*n* 15) document or content analysis (*n* 11), stakeholder interviews (*n* 8) or a combination of both document analysis and interviews (*n* 10). Two studies used stakeholder mapping. The studies covered seven broad food policy topics: national food and nutrition policies (*n* 9); non-communicable diseases and obesity (*n* 7); food and nutrition security (*n* 7); regulation and taxes (*n* 8); agri-food policy (*n* 4); urban food policy (*n* 4); sustainable food systems (*n* 4) and social justice (*n* 1).

Most studies analysed national-level public policymaking (*n* 33), with the remainder focused on local or regional policymaking processes. Geographically, studies were conducted in Europe (*n* 14), Oceania (*n* 10), Africa (*n* 7), North America (*n* 6), South America (*n* 1), Asia (*n* 2) and cross-continental (*n* 4). The highest proportion of studies was conducted in Australia (16 %).

The analytical frameworks applied differed across most of the studies. In twenty-one^(26–46)^studies, the authors used a previously published framework, others used a combination of two or more previously published frameworks in their analysis (*n* 12)^(47–58)^ and the remaining authors (*n* 9) developed their own analytical frameworks^( 59–67)^.

### Summary of frameworks used to analyse public policymaking processes in the food policy domain

Since the review papers were selected based on early policy cycle stages^(22)^, the included frameworks focus primarily on agenda-setting and policy formulation. Four frameworks focus on the entire policy cycle from agenda-setting to policy evaluation^(19,30,51,68)^. These frameworks are flexible in their analysis and provide high-level analysis guidelines for researchers. The remaining frameworks focus on one or two stages of the policy process, providing guidelines for in-depth analysis of each stage. Eight frameworks focus specifically on the agenda-setting stage^(20,25,60,66,67,69–71)^. Ten frameworks target both the agenda-setting and policy formulation stages^(24,27,55,59,61,62,72–75)^, and two frameworks target both the policy formulation and policy implementation stages^(47,76)^. Where frameworks are designed to target more than one stage of the policy process, critique is focused on their analyses of the agenda-setting and policy formulation stages.

Of the twenty-four frameworks, twenty-one were primarily analytical frameworks, designed to support theoretical analyses of policy processes. The remaining three frameworks were more evaluative, providing criteria to assess whether key elements of the policymaking process are in place.

### How applicable are the frameworks to the analysis of integrated food systems approaches to policymaking

Two frameworks were rated as ‘full’ for all three criteria, ‘Evaluative Framework on Directionality and Reflexivity’^(63)^ and the ‘Policy Integration Framework’^(47)^. The remaining frameworks received ‘partial’ ratings for at least two criteria, often focusing in depth on a single aspect – stakeholders, policy integration or policy context and framing (Tables 3 and 4). The majority of frameworks (*n* 23) examined actors in the analysis of policymaking processes. Most frameworks (*n* 23) also included space for analysis of policy context and policy framing. Policy coherence and integration analysis were considered in 46 % (*n* 11) of frameworks.

**Table 3.** Characteristics of the studies included in the review including methods and analytical frameworks used

Reference	Setting	Methods	Framework(s) used	Research question	Topic
Ares *et al*. (2021)^(56)^	Uruguay	Case study	Advocacy Coalition Framework (ACF) Halls theory of policy learning	Impact of policy context	Regulation, taxing, labelling policies
Ashton *et al*. (2021)^(32)^	Australia	Document analysis	‘What is the problem represented to be?’ (WPR) Framework	Agenda-setting Stakeholder analysis	Regulation, taxing, labelling policies
Balarajan and Reich (2016)^(29)^	India	Stakeholder interviews	Kingdon’s Multiple Streams Framework	Impact of policy context Agenda-setting	Food and nutrition security
Bastian (2011)^(58)^	Australia	Document analysis	Ham and Hill’s theory of policy analysis ‘What is the problem represented to be?’ (WPR) Framework	Impact of policy context	National food and nutrition policy
Bell *et al*. (2021)^(41)^	Tonga	Case study	The Health Policy Triangle Kingdon’s Multiple Streams Framework (MSF)	Impact of policy context	NCD and obesity
Biesbroek and Candel (2020)^(47)^	Netherlands	Case study	The Policy Integration Framework Context Mechanism Outcome Model	Policy integration, coherence and coordination	Sustainable food systems
Brandon *et al*. (2020)^(36)^	Australia	Case study	The Health Policy Triangle	Policy governance	National food and nutrition policy
Caraher *et al*. (2013)^(50)^	Australia	Document analysis	The Health Policy Triangle Kingdon’s Multiple Streams Framework (MSF)	National food and nutrition policy development	National food and nutrition policy
Carey *et al*. (2016)^(53)^	Australia	Document analysis	The Health Policy Triangle Kingdon’s Multiple Streams Framework (MSF)	National food and nutrition policy development	National food and nutrition policy
Cervantes *et al*. (2021)^(59)^	Mexico	Document analysis and stakeholder interviews	Food Systems Policy Space Analysis Framework Advocacy Coalition Framework (ACF)	Policy integration, coherence and coordination	National food and nutrition policy
Clarke *et al*. (2021)^(77)^	Australia	Document analysis and stakeholder interviews	Advocacy Coalition Framework (ACF) Institutional and Analysis Framework (IADF) Kingdon’s Multiple Streams Framework (MSF)	Policy governance	NCD and obesity
Clarke *et al*. (2019)^(54)^	Australia	Document analysis and stakeholder interviews	Advocacy Coalition Framework (ACF) Kingdon’s Multiple Streams Framework (MSF)	Agenda-setting Impact of policy context	Regulation, taxing, labelling policies
Cromwell and Chintedza (2005)^(43)^	Southern Africa	Stakeholder mapping	Research and Policy in Development (RAPID) Framework	Impact of policy context	Food and nutrition security
de Boon *et al*. (2021)^(46)^	Multi EU	Document analysis	The Policy Mix Framework	Policy integration, coherence and coordination	Agri-food policy
Dodd *et al*. (2020)^(33)^	Pacific	Document analysis	Advocacy Coalition Framework (ACF)	Agenda-setting Impact of policy context Policy coherence	NCD and obesity
Doernberg *et al*. (2019)^(38)^	Germany	Document analysis and stakeholder interviews	The Public Policy Cycle Process Framework	Agenda-setting Impact of policy context	Urban food policy
Feindt *et al*. (2021)^(60)^	International	Document analysis	The Politicization/Depoliticization Policy Change (PDCP) Model	Impact of policy context	Agri-food policy
Fuster *et al*. (2021)^(34)^	Mexico, Chile	Case study	Kaleidoscope Model for Policy Change	Agenda-setting Impact of policy context	Regulation, taxing, labelling policies
Galli *et al*. (2020)^(61)^	Europe	Document analysis	‘A conceptual framework for policy transition’	Impact of policy context Agenda-setting	Sustainable food systems
Hagenaars *et al*. (2020)^(26)^	USA	Stakeholder interviews	The Health Policy Triangle	Impact of policy context Agenda-setting	Regulation, taxing, labelling policies
Hagenaars *et al*. (2021)^(42)^	Multi-country	Case study	The Health Policy Triangle	Agenda-setting Impact of policy context	Regulation, taxing, labelling policies
Harris (2019)^(44)^	Zambia	Document analysis and stakeholder interviews	Power Cube Framework	Stakeholder analysis	National food and nutrition policy
Hebinck *et al*. (2017)^(62)^	UK, Netherlands	Case study	Operationalizing Participation and Accountability within Participatory Governance Framework – author’s own	Participatory policy processes	Urban food policy
Hobbs *et al*. (2004)^(45)^	USA	Stakeholder mapping	Advocacy Coalition Framework (ACF)	Stakeholder analysis Agenda-setting	National food and nutrition policy
Juma *et al*. (2018)^(31)^	Multi-country Africa	Case study	The Health Policy Triangle	Impact of policy context Stakeholder analysis	NCD and obesity
Kugelberg *et al*. (2012)^(27)^	Slovenia	Stakeholder interviews	Kingdon’s Multiple Streams Framework (MSF)	National food and nutrition policy development	National food and nutrition policy
Kugelberg *et al*. (2021)^(63)^	Finland, Sweden	Case study	Evaluative Framework on Directionality and Reflexivity – author’s own	Agenda-setting	Sustainable food systems
Maughan *et al*. (2020)^(64)^	UK	Content analysis	A Framework to Support a Process of Reading for Social Justice- author’s own	policy discourses policy integration	Social justice
McInnes (2019)^(48)^	Canada	Content analysis	Food Systems Triangle Multi-Level Perspective (MLP) Framework	Participatory processes	Sustainable food systems
Milani-Bonab *et al*. (2023)^(37)^	Iran	Document analysis	The Policy Integration Framework	Policy integration, coherence and coordination	Food and nutrition security
Minotti *et al*. (2021)^(55)^	Rome	Document analysis	The Policy Integration Framework Governance innovation theory	Policy integration, coherence and coordination	Urban food policy
Minotti *et al*. (2022)^(40)^	Milan	Content analysis	The policy cycle of Bridgman and Davis	Policy integration, coherence and coordination	Urban food policy
Mockshell and Birner (2015)^(28)^	Uganda, Ghana	stakeholder interviews	Advocacy Coalition Framework (ACF)	Stakeholder analysis	Agri-food policy
Ngqangashe *et al*. (2021)^(65)^	Multi-country	Case study	Regulatory Governance Analytical Framework – author’s own	Agenda-setting Impact of policy context	Regulation, taxing, labelling policies
Nisbett and Barnett (2017)^(39)^	India	Stakeholder interviews	Enabling Environment Framework	Impact of policy context	Food and nutrition security
Ouedraogo *et al*. (2020)^(51)^	Burkina Faso	Stakeholder interviews	SUN multisectoral planning frameworks Public policy development process	Policy integration, coherence and coordination	Food and nutrition security
Pelletier *et al*. (2012)^(49)^	Bolivia, Peru, Guatemala and Vietnam	Stakeholder interviews	Shiffman’s Agenda-Setting Framework The Policy Sciences Framework	Impact of policy context food policy in developing countries	Food and nutrition security
Reeve *et al*. (2021)^(52)^	Solomon Islands	Document analysis and stakeholder interviews	Kingdon’s Multiple Streams Framework (MSF) Advocacy Coalition Framework (ACF) Policy cycle heuristic	Impact of policy context	Regulation, taxing, labelling policies
Resnick *et al*. (2018)^(30)^	Zambia	Case study	Kaleidoscope Model for Policy Change	Impact of policy context	Food and nutrition security
Sisnowski *et al*. (2016)^(57)^	USA	Case study	Kingdon’s Multiple Streams Framework (MSF) International Obesity Task Forces evidence-based decision-making framework	Impact of policy context	NCD and obesity
Studlar and Cairney (2019)^(66)^	Multi-country	Case study	Agenda-setting, Policymaking environment and Windows of opportunity Framework – author’s own	Impact of policy context	NCD and obesity
Thow *et al*. (2014)^(35)^	Ghana	Stakeholder interviews	The Health Policy Triangle	Policy integration, coherence and coordination	NCD and obesity
Timotijevic *et al*. (2013)^(67)^	Europe	Case study	EURRECA – Public Health Nutrition Policy-making Framework	Agenda-setting Evidence to policy	National food and nutrition policy

NCD, non-communicable disease; SUN, Scaling Up Nutrition Initiative; EURRECA, European Registries for Rare Endocrine Conditions.

**Table 4.**
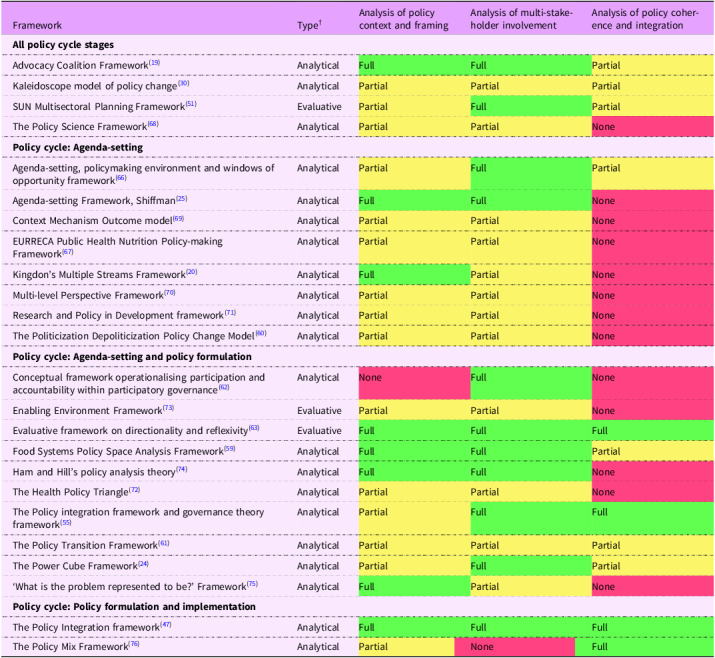
Assessment of policy frameworks against criteria^*^ for ‘food systems’ approaches to policymaking

SUN, Scaling Up Nutrition Initiative; EURRECA, European Registries for Rare Endocrine Conditions. ‘Full’ indicates a framework that facilitates a comprehensive food systems analysis of the criterion. ‘Partial’ indicates a framework that includes a prompt for the criterion analysis but less comprehensive in terms of ‘food systems’ analysis. ‘None’ indicates a framework that does not include a prompt for the criterion.

^†^ Framework types are defined as analytical: *designed to support theoretical analyses of policy processes; and evaluative, providing criteria to assess whether key elements of the policymaking process are in place.*

* Criteria are based on definitions of integrated food policy^(23)^ and recommendations for integrated food policymaking in the Organisation for Economic Co-operation and Development (OECD)‘Making Better Policies for Food Systems’ report^(11)^. Frameworks are categorised based on the stage(s) of the policy cycle they target^(22)^.

The following sections provide details on how each criterion was approached in the frameworks.

### Criterion 1: Analysis of policy context and framing

Theories of agenda-setting analysis consist of understanding the policy context, economic conditions and resource provision, norm promotion and focusing events, all leading to policy windows. Most frameworks included an element of these theories. For example, policy frameworks such as the ‘Health Policy Triangle’^(72)^ and the ‘Policy Science Framework’^(68)^ prompt consideration of policy context but are largely considered more descriptive frameworks and leave the interpretation and depth of analysis open to the researcher. Ham and Hill’s policy analysis framework^(74)^ and the ‘Food Systems Policy Space Analysis Framework’^(59)^ are more detailed and specify the need to analyse the economic, political, social and cultural circumstances that lead to policy development. Though not specific to food systems policies, these frameworks can be applied to most policymaking analyses.

Of the frameworks with a specific focus on agenda-setting, three were rated ‘full’. Shiffman’s ‘Agenda-setting Framework’^(25)^ provides an in-depth structure for analysis of policy context, ideas, actor power and policy issue characteristics. The Kugelberg *et al*. ‘framework to increase government capacity for directionality and reflexivity to support integrated food policy’^(63)^ specifically examines whether agenda-setting tools within policymaking processes consider food systems approaches. In the context of food systems policymaking processes, an understanding of how policies and policy problems are framed at the agenda-setting stage can give insight into whether a need for a ‘whole-of-government’ or ‘systems’ approach is considered important from the outset. The ‘Enabling Environment Framework’^(73)^ focuses on the ‘*framing, generation and communication of knowledge and evidence*’ and the ‘*political economy of stakeholders, ideas and interests*’ and provides specific indicators of analysis. Grounded in a ‘nutrition in all policies’ perspective, this framework holds potential application in food systems research.

Five frameworks have a focus on policy problem framing specifically. The ‘Policy Integration Framework^(47)^’, the ‘Policy Transition Framework’^(61)^ and the ‘Scaling Up Nutrition (SUN) Multisectoral Framework’^(57)^ are particularly relevant to food policymaking processes. The ‘Policy Integration Framework’ prompts researchers to examine the ‘*extent to which policy problems are recognised as “cross-cutting”*’ and requiring a holistic governance approach. The ‘SUN Multisectoral Framework’^(51)^ similarly assesses the policy problem framing in terms of how the problem is defined by different stakeholders across sectors and the impact this has on policymaking approaches. A similar approach is taken in Bacchi’s ‘What is the problem represented to be?’ policy framework^(75)^. The ‘Policy Transition Framework’^(61)^ puts policy framing at the centre of the analysis of policy processes for sustainable food systems. It considers the impact of policy framing, consisting of narratives and representations of food systems, on sustainable food policy spaces, objectives and instruments.

### Criterion 2: Analysis of multi-stakeholder involvement

While all frameworks include elements for actor analysis, the depth of analysis they allow varies. Most of the frameworks include space for listing the actors involved in the policy process. However, in the context of food systems approaches to policymaking, it is important to go beyond this and understand the relationships between the actors involved and how they are included within the process^(78)^. Seven frameworks allow for a thorough analysis of how actors are included in the policy process. These frameworks aim to answer questions such as ‘*Do all of the necessary stakeholders have a seat at the table?*’, ‘*How inclusionary were the engagement processes?*’ and ‘*Who holds the power in the policy process?*’.

Most relevant to a food systems approach is the Kugelberg *et al*. ‘framework to increase government capacity for directionality and reflexivity to support integrated food policy’^(63)^. This framework includes criteria, specific to food policymaking, to evaluate the ‘*structure of whole-of-government approaches*’, ‘*style of engagement process*’ and ‘*nature of multi-stakeholder evaluations*’. Under each criterion, it offers examples of policymaking structures and strategies that are optimal for food systems approaches and those that meet the basic requirements for food systems approaches, ranked on a four-point scale. For example, under the ‘*structure of whole-of-government approaches*’ criterion, ‘*A formal platform, chaired by the highest authority, e.g Prime Minister, involving most ministries to set a future vision of the food system*’ is given as an example of an optimal approach. The inclusion of food policy-specific indicators in the framework offers a clear benchmarking tool for food policy researchers and policymakers. Hebinck *et al*.’s ‘conceptual framework operationalising participation and accountability within participatory governance’^(62)^ also analyses the nature of stakeholder engagement processes. It asks, ‘*Are those that have a stake invited to the table?*’ and allows assessment of the quality of participatory approaches.

Understanding the distribution of power within stakeholder networks is also important for analysing food policymaking processes. The concept of stakeholder power dynamics is featured in Ham and Hill’s^(74)^ and Studlar and Cairney’s policy analysis frameworks^(66)^. Both frameworks call for the consideration of power in policy analyses but lack specificity on how the analysis should be conducted. The ‘Power Cube Framework’^(24)^ was developed specifically for the analysis of power within policy processes. It enables examination of the forms of power held by stakeholders such as visible power in decision-making, hidden power in agenda-setting or invisible power in establishing political and societal norms. It also includes the analysis of power ‘spaces’, describing the opportunities for stakeholders to engage in the process, and levels of power at international, national and local political levels.

The final two frameworks, the ‘Policy Mix Framework’^(76)^ and the ‘Policy Integration Framework’^(47)^, focus on policy formulation and implementation and also enable assessment of actors and their roles within the policymaking process. The ‘Policy Integration Framework’^(47)^ includes a question on the ‘*range of actors and institutions involved in the governance of a cross-cutting policy problem*’ and the ‘*density of interactions*’ between them. However, the ‘Policy Mix Framework’^(76)^ asks about ‘*the range of actors*’ involved in the policy process, their roles and the style of engagement. These frameworks are less prescriptive than others, affording researchers more analytical flexibility but requiring a more unguided, intuitive analysis, which may present challenges for those with limited expertise in policy theory.

### Criterion 3: Analysis of policy coherence and integration

Policy integration is a key feature of food systems policies. A comprehensive analysis of how ‘integrated’ a policy is requires an assessment of horizontal and vertical coherence between government policy goals and between actors inside and outside of government^(23)^. Five of the twenty-four frameworks include space for a comprehensive assessment of how joined-up or integrated policy approaches are considered within the policymaking process.

The ‘Policy Mix Framework’^(76)^ aims to analyse the development and coherence of policy mixes. It addresses the existence or absence of trade-offs between policies and the horizontal and vertical coherence between different policy goals. This is the only framework to specifically address coherence between different levels of governance, an important feature of food systems policies. The ‘Policy Integration Framework’^(47)^ and the ‘Policy Integration and Governance Theory Framework’^(55)^ include an analysis of policy goals (the range of policies that address a cross-cutting problem and the subsequent coherence between those policy goals) and policy instruments (consistency of policy instrument mixes and the presence of policy instruments to coordinate policy efforts at a systems level). The focus in this framework is primarily on horizontal coherence across government sectors, but there is space to include analysis of vertical coherence between government levels and between actors internal and external to government.

In the directionality and reflexivity framework^(63)^, the analysis is approached through assessing the presence of systems that allow for the consideration of policy coherence. The section ‘*scope and objectives of prior assessments*’ asks whether prior assessments (e.g. impact assessments, foresight studies and research studies) address trade-offs and incoherences in previous policy goals. The section on ‘*the role of multi-stakeholder platforms*’ addresses the opportunities for stakeholders across sectors to engage in debate on the policy objectives and highlight incoherences.

## Discussion

The aim of this review was to summarise the political science frameworks used in the literature to analyse public policymaking processes in the food policy domain and to assess how applicable the identified frameworks are to analysing integrated food systems approaches in policymaking.

Only 10 % of the review studies used explicit analytical frameworks to analyse policymaking processes. Similar to previous reviews^(16,17)^, we found that well-established frameworks such as ‘Kingdon’s Multiple Streams Theory’^(20)^, the ‘Advocacy Coalition Framework’^(19)^ and Health Policy Triangle’ framework^(72)^ were most frequently used. While previous reviews examined how political science theories were applied in public health research^(13,16–18)^, in the context of food policy research, an assessment of whether these frameworks allowed for a comprehensive assessment of the policymaking activities central to food systems approaches was missing from the literature.

Using criteria based on OECD recommendations for better food systems policies^(11)^, we assessed twenty-four frameworks previously applied in food policy research on how comprehensively they could assess whether policymaking processes are conducive to integrated food systems policies. Most of the frameworks included at least one relevant parameter relating to the criteria. These included analysis of policy context and policy framing, the nature of stakeholder involvement and influence and policy coherence and integration. Many studies in this review used a combination of frameworks in their analysis or combined components of multiple frameworks to fill gaps and suit their research questions^(17,33,41,47–54,56,58,59,66)^. Additionally, some frameworks could be applied to all stages of the policy cycle, while others focused primarily on the first two or three stages.

In their application to food systems policy research and the evaluation of integrated food systems approaches to policymaking, the Kugelberg *et al*. ‘Evaluative Framework on Directionality and Reflexivity’^(63)^ and the ‘Policy Integration Framework’^(47)^ are the most comprehensive and provide the basis for extensive evaluation and analysis of multi-stakeholder involvement, policy framing and policy coherence and integration. These are also the only frameworks that fully consider both horizontal and vertical coherence and the integration of food policies in the policymaking process, an essential component of integrated food systems policy^(23)^. Both frameworks can also be applied to different stages of the policy cycle. The frameworks have been applied by the authors to compare food policymaking processes in Finland and Sweden^(63)^ and to examine policy (dis)integration in the Netherlands^(47)^.

In our analysis, we identified a distinction between analytical and evaluative frameworks previously used in the food policy domain. While the majority of frameworks reviewed were analytical and designed to support in-depth theoretical analyses of the policy process, three frameworks were more evaluative and offered criteria to assess whether key elements of an integrated food policy process were present. Two of these frameworks, the ‘SUN Multisectoral Framework’^(51)^ and the ‘Enabling Environment Framework’^(73)^, were developed specifically to facilitate coordinated cross-sectoral policy development for nutrition and provide evaluation criteria by identifying and addressing the necessary conditions for effective policy development and implementation. The Evaluative Framework on Directionality and Reflexivity’^(63)^, focusing on agenda-setting and policy formulation, was also developed as a tool for policymakers and identifies the necessary governance structures for a systems approach to food policymaking. Within each identified criterion, it uses a four-point scale to provide examples of optimal strategies and structures for policy development, providing a practical benchmarking tool for policymakers.

This distinction between frameworks reflects the frequently discussed differences between analysis *of* policy and analysis *for* policy and the trade-offs between theoretical richness and practical usability^(79)^. Analytical frameworks are generally more suited to academic research and generating an in-depth understanding of policymaking dynamics such as path dependency or institutional constraints but are often more intuitive and lack the operational clarity required by policymakers to guide policymaking.

The ‘Policy Integration Framework’^(47)^ is an analytical framework focusing on policy formulation and integration. While not specifically targeted towards food policy, it includes all of the criteria relevant to systems approaches to food policymaking. It also provides specific analytical questions with detailed direction on their application, useful for researchers outside the political science domain. Another highly rated analytical framework specifically targeted at food systems and policy analysis is that of Cervantes *et al*.^(59)^. Focusing on agenda-setting and policy formulation, the authors combine policy space analysis theory, the ACF framework^(19)^ and the food supply chain stages to create their framework for analysis and apply it to examine the integration of nutrition in Mexican food policy. While this framework misses an analysis of policy coherence, it provides the basis for a strong analysis, through a food systems lens, of policy context, agenda-setting and the impact of actors on the policy process.

In food systems approaches to policymaking, which require coordination across multiple sectors and governance levels, both evaluative and analytical frameworks can prove valuable tools to creating more integrated and coherent policymaking: by aiding policymakers in identifying gaps and (mis)alignment issues in current processes and allowing researchers to unpack complex system dynamics.

### Limitations

The review has a number of limitations. First, only two databases were searched, and combined with limitations in the indexing of qualitative research terms, this may have resulted in an incomplete retrieval of relevant studies^(80)^. To address this, only highly relevant databases were selected, and publications from all years were included during the search. The number of final studies included in the review was similar to or exceeded previous reviews^(13,17,18)^. Similarly, though the search for analytical frameworks was not exhaustive, a comparatively high number of frameworks were identified. These frameworks were often used in multiple studies, suggesting that the most prominent frameworks used in food policy research were identified. Another limitation is that only one author (IS) was involved in the search process, the data charting process and the evaluation of the frameworks (the search strategy was reviewed by a research librarian, and the second author advised on inclusion criteria throughout the screening process). A second reviewer could have strengthened the review process and increased the robustness of the analysis, though this is not always agreed on in qualitative research settings^(81)^.

### Conclusions

This review assessed the application of political science frameworks in the analysis of food policymaking processes and their applicability in food systems research. Though the use of analytical frameworks to analyse policymaking processes is limited in the food policy field, this review demonstrates that there are benefits for their use in understanding the ideas, knowledge, power and decision-making that lead to food policy development. This is particularly true in the context of policymaking for integrated and coherent food policies for sustainable food systems, where policy success relies heavily on the meaningful engagement of stakeholders, holistic policy framing and consideration of related policy goals and objectives. We identified two frameworks in particular that provide comprehensive analytical criteria for such analyses and have been successfully applied to the analysis of food policymaking processes using a food systems lens. Future research could apply the same frameworks in different contexts to build a comparable body of evidence for considering the impact of political processes on integrated food systems policies. This review forms the basis of a wider programme of work aiming to conduct such analyses and contribute to the application of political science methodology for understanding food systems approaches to public policymaking.

## Supporting information

Stanley and Murrin supplementary material Stanley and Murrin supplementary material

## Supplementary material

For supplementary material accompanying this paper, visit https://doi.org/10.1017/S1368980025100906.
