# Supplementary material for: Analysis of food policymaking through a food systems lens: a review of analytical frameworks
Source: Public Health Nutr. 2025 Aug 12;28(1):e158. doi: 10.1017/S1368980025100906 (PMC12516611; doi:10.1017/S1368980025100906)
Supplement: Stanley and Murrin supplementary material [file S1368980025100906sup001.docx]

| Table S1. Evaluation of policy analysis frameworks for assessing integrated food systems policymaking processes. | | | |
| --- | --- | --- | --- |
| **Framework** | **Criterion** | **Rating** | **Justification** |
| Advocacy Coalition Framework ^(1)^ | Policy Context and Framing | Full | Strongly addresses policy context, including political systems, sociocultural values and external subsystem events that influence policy development. Also addresses policy framing in describing the attributes of the policy problem and examining policy core beliefs and actor beliefs. |
|  | Multi-Stakeholder Involvement | Full | Focuses on diverse actor coalitions, allows for the identification of a wide range of actors across levels and types of government, provides robust analysis of stakeholder interactions, values, and roles and analyses the relationship between actor power and ideas. |
|  | Policy Coherence and Integration | Partial | Examines policy interactions across policy subsystems but does not fully address horizontal and vertical policy alignment or integration across governance levels. |
| Kaleidoscope model of policy change ^(2)^ | Policy Context and Framing | Partial | Examines how policy problems are framed and how norms, values and beliefs impact agenda setting. Considers context and political economy but lacks explicit systems framing or emphasis on cross-sectoral integration. |
|  | Multi-Stakeholder Involvement | Partial | Recognises political actors and the impact of power distribution on the policymaking process but does not deeply explore stakeholder diversity, power dynamics, or engagement processes. |
|  | Policy Coherence and Integration | Partial | Touches on policy feedback and alignment, but lacks comprehensive tools to assess integration or coherence across sectors and levels. |
| SUN Multisectoral Planning Framework^(3)^ | Policy Context and Framing | Partial | Recognises the need for multisectoral action and evaluates the inclusion of comprehensive situational analyses in the national nutrition plans but lacks consideration of policy problem framing and cross-sectoral framing of food systems problems. |
|  | Multi-Stakeholder Involvement | Full | Examines multi-sector and multi-stakeholder engagement in the development of national nutrition plans. It also considers stakeholder interaction and roles in the form of governance, accountability and coordination mechanisms. |
|  | Policy Coherence and Integration | Partial | Emphasises multisectoral alignment and coherent policy planning and assesses the recognition of cross-sectoral policy links and coherence within national nutrition policymaking processes. Does not explicitly consider policy integration mechanisms or vertical coherence. |
| *SUN, Scaling Up Nutrition Initiative*. ‘Full’ indicates a framework that facilitates a comprehensive food systems analysis of the criterion. ‘Partial’ indicates a framework that includes a prompt for the criterion analysis but less comprehensive in terms of ‘food systems’ analysis. ‘None’ indicates a framework that does not include a prompt for the criterion. *Criteria are based on definitions of integrated food policy^(4)^ and recommendations for integrated food policymaking in the Organisation for Economic Co-operation and Development (OECD )‘Making Better Policies for Food Systems’ report^(5).^ | | | |

| Table S1 continued. Evaluation of policy analysis frameworks for assessing integrated food systems policymaking processes. | | | |
| --- | --- | --- | --- |
| **Framework** | **Criterion** | **Rating** | **Justification** |
| The Policy Science Framework ^(6)^ | Policy Context and Framing | Full | Facilitates analysis of policy framing and policy issue characteristics in the 'problem orientation' dimension and enables examination of the impact of actor beliefs on policy framing and narratives on policy processes. Includes strong analysis of economic, political and social contexts in which the policy is made. Does not explicitly approach the analysis using a systems perspective but all analysis elements are present. |
|  | Multi-Stakeholder Involvement | Full | Allows for the mapping of all stakeholders, roles and beliefs and the quality of stakeholder engagement in the policy process. Also prompts analysis of stakeholder interaction, strategies and power through influence and social roles. |
|  | Policy Coherence and Integration | None | Implicitly considers trade-offs and spillovers through the 'problem orientation' dimension and encourages analysis of policy interconnections across domains but lacks explicit analysis of policy coherence and integration. |
| Agenda setting , policymaking environment and windows of opportunity framework ^(7)^ | Policy Context and Framing | Partial | Considers some aspects of the policymaking environment and agenda-setting including policy framing, socio-economic context (public support, prevalence and economic impacts) and ideas and scientific evidence but does not apply a full systems approach. |
|  | Multi-Stakeholder Involvement | Full | Allows for the analysis of actors across government levels, policy networks and power dynamics, institutional responsibilities for policy and actor ideas and beliefs. |
|  | Policy Coherence and Integration | Partial | Considers the presence of actors at different government levels and coherence with international policies but lacks many elements needed for an in-depth analysis of policy coherence and integration. |
| Agenda-setting Framework, Shiffman^(8)^ | Policy Context and Framing | Partial | Examines how policy problems are framed, including actor ideas and narrative strategies and the political and social contexts that determine issue prioritization and form policy windows. |
|  | Multi-Stakeholder Involvement | Full | Facilitates analysis of actors, roles and the degree of coalescence between individuals and organisations. Also examines the effectiveness of coordination mechanisms and the strength of the actors concerned with the issue. |
|  | Policy Coherence and Integration | None | Does not consider policy coherence or integration. |
| ‘Full’ indicates a framework that facilitates a comprehensive food systems analysis of the criterion. ‘Partial’ indicates a framework that includes a prompt for the criterion analysis but less comprehensive in terms of ‘food systems’ analysis. ‘None’ indicates a framework that does not include a prompt for the criterion.*Criteria are based on definitions of integrated food policy^(4)^ and recommendations for integrated food policymaking in the Organisation for Economic Co-operation and Development (OECD )‘Making Better Policies for Food Systems’ report(^5)^. | | | |

| Table S1 continued. Evaluation of policy analysis frameworks for assessing integrated food systems policymaking processes. | | | |
| --- | --- | --- | --- |
| **Framework** | **Criterion** | **Rating** | **Justification** |
| Context Mechanism Outcome model^(9)^ | Policy Context and Framing | Partial | Considers policy context in a causal explanatory way, but not from a systems lens. Missing an analysis of policy framing. |
|  | Multi-Stakeholder Involvement | Partial | Recognises actor involvement in mechanisms but lacks emphasis on engagement quality, power and stakeholder dynamics. |
|  | Policy Coherence and Integration | None | May consider outcome alignment, but coherence or integration across policy domains is not explicitly analysed. |
| EURRECA Public Health Nutrition Policy-making Framework^(10)^ | Policy Context and Framing | Partial | Includes consideration of public health policy context, including actor beliefs and global trends but lacks comprehensive systemic framing or political analysis. |
|  | Multi-Stakeholder Involvement | Partial | Identifies relevant actors but does not fully analyse their engagement, interrelationships or power dynamics. |
|  | Policy Coherence and Integration | None | Offers no guidance for analysing policy coherence, trade-offs, spillovers, or integration across governance levels. |
| Kingdon's Multiple Streams Framework^(11)^ | Policy Context and Framing | Full | Facilitates a comprehensive analysis of agenda-setting including issue framing and policy context (focusing events, external and global influences). |
|  | Multi-Stakeholder Involvement | Partial | Provides an assessment of the roles of multiple actors (guiding institutions, policy advocates and interest groups) and the alignment of stakeholders on policy issues but does not consider quality of stakeholder engagement in the policy process or an assessment of power dynamics. |
|  | Policy Coherence and Integration | None | Considers the alignment of stakeholder beliefs and the impact of global policies and agendas but does not provide an analysis of policy coherence and integration. |
| *EURRECA, European Registries for Rare Endocrine Conditions*. ‘Full’ indicates a framework that facilitates a comprehensive food systems analysis of the criterion. ‘Partial’ indicates a framework that includes a prompt for the criterion analysis but less comprehensive in terms of ‘food systems’ analysis. ‘None’ indicates a framework that does not include a prompt for the criterion.*Criteria are based on definitions of integrated food policy^(4)^ and recommendations for integrated food policymaking in the Organisation for Economic Co-operation and Development (OECD )‘Making Better Policies for Food Systems’ report^(5)^. | | | |

| Table S1 continued. Evaluation of policy analysis frameworks for assessing integrated food systems policymaking processes. | | | |
| --- | --- | --- | --- |
| **Framework** | **Criterion** | **Rating** | **Justification** |
| Multi-level Perspective Framework^(12)^ | Policy Context and Framing | Partial | Strong on understanding the policy landscape and political, environmental and social contexts (environment, demographic trends, political ideology, societal values and economic patterns). Included some consideration of policy framing but does not fully assess policy context and framing from a systems perspective. |
|  | Multi-Stakeholder Involvement | Partial | Considers the roles of niche actors and institutions but does not prompt analysis of stakeholder dynamics, power or assess stakeholder engagement. |
|  | Policy Coherence and Integration | None | Examines policy interactions at the 'regime' level in the context of the impact of past policies and regulations but does not allow for policy coherence or integration analysis. |
| Research and Policy in Development framework^(13)^ | Policy Context and Framing | Partial | Examines the political context of policies, the prevailing narratives surrounding a policy and actor knowledge and priorities in policy development but focuses mainly on the link between evidence and policy and lacks a systems perspective. |
|  | Multi-Stakeholder Involvement | Partial | Facilitates a comprehensive analysis of actor roles, networks and power within the policy processes including stakeholder influence and priorities but the analytical focus is on the evidence-policy link and analysis of the quality of stakeholder engagement is missing. |
|  | Policy Coherence and Integration | None | Does not consider policy coherence or integration. |
| The Politicization Depoliticization Policy Change Model^(14)^ | Policy Context and Framing | Partial | Explores the framing of issues in political agendas, including how they are strategically constructed to align with political interests. Does not fully consider policy context or contextual multi-sectoral factors influencing the policymaking process |
|  | Multi-Stakeholder Involvement | Partial | Focuses analysis of elite and key political actor beliefs and roles in decision making processes but , lacks comprehensive analysis of diverse stakeholder groups and their roles, stakeholder engagement in the policy process and power dynamics. |
|  | Policy Coherence and Integration | None | Does not analyze the coherence between different policy goals, trade-offs, or alignment across sectors and governance levels. |
| ‘Full’ indicates a framework that facilitates a comprehensive food systems analysis of the criterion. ‘Partial’ indicates a framework that includes a prompt for the criterion analysis but less comprehensive in terms of ‘food systems’ analysis. ‘None’ indicates a framework that does not include a prompt for the criterion.*Criteria are based on definitions of integrated food policy^(4)^ and recommendations for integrated food policymaking in the Organisation for Economic Co-operation and Development (OECD )‘Making Better Policies for Food Systems’ report^(5)^. | | | |

| Table S1 continued. Evaluation of policy analysis frameworks for assessing integrated food systems policymaking processes. | | | |
| --- | --- | --- | --- |
| **Framework** | **Criterion** | **Rating** | **Justification** |
| Conceptual framework operationalizing participation and accountability within participatory governance^(15)^ | Policy Context and Framing | None | Explores the impact of the policy environment on how the participatory process is structured but does not provide a comprehensive analysis of policy context and does not consider policy framing from a systems perspective. |
|  | Multi-Stakeholder Involvement | Full | Facilitates an in-depth analysis of participatory policy processes and stakeholder involvement, focusing on participation and accountability. Examines the types of stakeholders and their relationships, quality of engagement and whether all who have a 'stake' have a seat at the table. |
|  | Policy Coherence and Integration | None | Provides space for the consideration of the potential negative impacts of policy actions but does not provide a sufficient analysis of coherence and integration. |
| Enabling Environment Framework^(16)^ | Policy Context and Framing | Partial | Prompts analysis of policy framing and narratives and problem definition using a cross-sectoral lens. Considers policy context through political structures, resources and knowledge generation and evidence. |
|  | Multi-Stakeholder Involvement | Partial | Examines multi-stakeholder platforms and participation structures including coalitions, leadership and institutions. Lacks a full analysis of stakeholder roles, engagement and power dynamics. |
|  | Policy Coherence and Integration | Partial | Considers coherence and alignment of institutions in terms of political commitment and resource alignment. Examines presence of structures within the policy process to support horizontal and vertical policy coherence. Does not explicitly address mechanisms for assessing tradeoffs and spillovers or policy instrument level coherence and integration. |
| Evaluative framework on directionality and reflexivity^(17)^ | Policy Context and Framing | Full | Assesses food policy processes using a food systems lens. Provides benchmarks for policy framing from a systems perspective and consideration of cross-sectoral perspectives in prior assessments and 'whole-of-government' approaches to the policy process. |
|  | Multi-Stakeholder Involvement | Full | Examines the structure 'whole-of-government' approaches and provides benchmarks for the structure of multi-stakeholder platforms, style and quality of engagement processes and the power multi-actor platforms have within the policy process. |
|  | Policy Coherence and Integration | Full | Facilitates an analysis of whether policy trade-offs, synergies and unintended policy impacts are considered from the beginning of the policy process and in policy impact evaluations, including horizontal and vertical coherence between policies. Policy integration is examined through the analysis of multi-stakeholder approaches. |
| ‘Full’ indicates a framework that facilitates a comprehensive food systems analysis of the criterion. ‘Partial’ indicates a framework that includes a prompt for the criterion analysis but less comprehensive in terms of ‘food systems’ analysis. ‘None’ indicates a framework that does not include a prompt for the criterion.*Criteria are based on definitions of integrated food policy^(4)^ and recommendations for integrated food policymaking in the Organisation for Economic Co-operation and Development (OECD )‘Making Better Policies for Food Systems’ report^(5)^. | | | |

| Table S1 continued. Evaluation of policy analysis frameworks for assessing integrated food systems policymaking processes. | | | |
| --- | --- | --- | --- |
| **Framework** | **Criterion** | **Rating** | **Justification** |
| Food Systems Policy Space Analysis Framework ^(18)^ | Policy Context and Framing | Full | Provides a policy space analysis including policy context (political, social and economic), agenda-setting circumstances (policy framing and perceptions of stakeholders) and policy characteristics through a food systems lens. |
|  | Multi-Stakeholder Involvement | Full | The framework incorporates elements of the Advocacy Coalition Framework to conduct an analysis of multi-stakeholder involvement including roles of actors across the food system and power dynamics. |
|  | Policy Coherence and Integration | Partial | The framework has a strong recognition of interconnections across the food system and explores policy interactions and coherence across food and nutrition related policies but lacks a full analysis of horizontal and vertical coherence and integration. |
| Ham and Hill’s policy analysis theory^(19)^ | Policy Context and Framing | Partial | Facilitates an in-depth analysis of policy context , focusing on the systemic facors that shape policy options including economic and social structures and the role of the state. It also allows for the examination of how policies are framed and how and why policy decisions are made. It does not fully consider cross-sectoral analysis of policy context factors. |
|  | Multi-Stakeholder Involvement | Full | Provides an in-depth analysis of multi-stakeholder involvement examining structural power imbalances, institutional mechanisms of engagement (who is invited and who participates) and stakeholder beliefs and values in decision making processes. |
|  | Policy Coherence and Integration | None | Framework could be used to understand political or structural barriers to policy coherence or integration but is not designed for the analysis of policy coherence or integration in policy processes. |
| The Health Policy Triangle ^(20)^ | Policy Context and Framing | Partial | Allows for the analysis of policy context and broader environment that shapes policy spaces. Includes an analysis of the policy process including how policies are initiated and agenda-setting. Lacks a systems framing perspective and a full analysis of the influence of policy problem framing on policymaking. |
|  | Multi-Stakeholder Involvement | Partial | Prompts the analysis of actor and stakeholder roles and some analysis of how actor interests and power shape the policy process but with limited focus on engagement quality or inter-actor dynamics. |
|  | Policy Coherence and Integration | None | Limited focus on coherence or integration beyond structural dimensions of policy. |
| ‘Full’ indicates a framework that facilitates a comprehensive food systems analysis of the criterion. ‘Partial’ indicates a framework that includes a prompt for the criterion analysis but less comprehensive in terms of ‘food systems’ analysis. ‘None’ indicates a framework that does not include a prompt for the criterion. *Criteria are based on definitions of integrated food policy^(4)^ and recommendations for integrated food policymaking in the Organisation for Economic Co-operation and Development (OECD )‘Making Better Policies for Food Systems’ report^(5)^. | | | |

| Table S1 continued. Evaluation of policy analysis frameworks for assessing integrated food systems policymaking processes. | | | |
| --- | --- | --- | --- |
| **Framework** | **Criterion** | **Rating** | **Justification** |
| The Policy integration framework and governance theory framework^(21)^ | Policy Context and Framing | Partial | Facilitates analysis of policy framing and how the problem is understood in the system. The framework is underpinned by a food systems lens and has an explicit focus on whether the cross-sectoral nature of the problem is recognised. Some consideration of context in terms of needs and problems in the population but does not prompt full analysis of wider contextual factors. |
|  | Multi-Stakeholder Involvement | Full | Provides a comprehensive analysis of policy subsystems; the range of actors and institutions involved in governance, density of interactions between them and where the political initiative lies. |
|  | Policy Coherence and Integration | Full | Facilitates analysis of policy goals and objectives and alignment goals across subsystems and domains. Also analyses policy instruments and how they are used to modify the political process to ensure coordination and integration. |
| Conceptual Framework for Policy Transition^(22)^ | Policy Context and Framing | Partial | Facilitates analysis of policy framing through a food systems lens, including identification of dominant and alternative policy frames, and how narratives can differ across sectors and how they shape policy actions and impacts. It also allows for the analysis of the broader policy landscape in the form of socio-technical regimes and prompts analysis of agenda-setting though does not fully consider cross-sectoral analysis of policy context. |
|  | Multi-Stakeholder Involvement | Partial | Allows for the consideration of actor values and beliefs in shaping policy frames and narratives but does not enable a comprehensive analysis of stakeholder roles, engagement and power dynamics. |
|  | Policy Coherence and Integration | Partial | Provides prompts for the consideration of trade-offs and coherence between policy goals in policy mixes at the policy design stage but does not allow for a full analysis of policy coherence or integration from a systems perspective. |
| The Power Cube Framework ^(23)^ | Policy Context and Framing | Partial | Examines policy context in relation to the types of decision-making spaces present in the policymaking process but does not facilitate a 'systems' analysis of the contextual factors shaping policy development or how the policy is framed. |
|  | Multi-Stakeholder Involvement | Full | Facilitates a comprehensive analysis of stakeholder involvement through the lens of a power analysis. Examines the 'spaces for participation', 'places and levels for participation' and the 'forms and visibility of power across spaces and places'. |
|  | Policy Coherence and Integration | Partial | Includes an examination of power dynamics at different levels of governance (global, national and local) and their impact on participatory policy approaches at other governance levels. It does not allow for an analysis of policy coherence. |
| Full’ indicates a framework that facilitates a comprehensive food systems analysis of the criterion. ‘Partial’ indicates a framework that includes a prompt for the criterion analysis but less comprehensive in terms of ‘food systems’ analysis. ‘None’ indicates a framework that does not include a prompt for the criterion. *Criteria are based on definitions of integrated food policy^(4)^ and recommendations for integrated food policymaking in the Organisation for Economic Co-operation and Development (OECD )‘Making Better Policies for Food Systems’ report(^5)^. | | | |

| Table S1 continued. Evaluation of policy analysis frameworks for assessing integrated food systems policymaking processes. | | | |
| --- | --- | --- | --- |
| **Framework** | **Criterion** | **Rating** | **Justification** |
| ‘What is the problem represented to be?’ Framework^(23)^ | Policy Context and Framing | Full | Facilitates an in-depth analysis of policy context and framing though understanding how a policy problem represented by different actors, the assumptions and political and social contexts underlying the representation and the impact of the representation on the policy process and action outcomes. |
|  | Multi-Stakeholder Involvement | Partial | Considers stakeholder values and beliefs in relation to policy problem definition and considers the impact of power and influence on how policy problems are defined and for what reason. Analysis of stakeholder involvement in the policy process and a more comprehensive assessment of power is missing. |
|  | Policy Coherence and Integration | None | Considers how the problem framing might have unintended consequences for different stakeholders/actors but does not allow for analysis of policy coherence or integration |
| The Policy Integration Framework^(24)^ | Policy Context and Framing | Full | Explicitly focuses on cross-sectoral, integrated framing of policy issues from a systems perspective; how cross-cutting policy problems are recognized by actors and institutions and the impact of wider political context and structures. |
|  | Multi-Stakeholder Involvement | Full | Evaluates the involvement of multiple sectors, governance levels and subsystem structures; the range of actors and institutions involved and the density of interactions between them. |
|  | Policy Coherence and Integration | Full | Provides comprehensive analysis of horizontal and vertical policy integration, including trade-offs, spillovers, and alignment of policy objectives and goals across sectors. |
| The Policy Mix Framework^(25)^ | Policy Context and Framing | Partial | Addresses policy framing in the context of policy strategies and rationales and in the analysis of policymaking processes but lacks a direct analysis of the broader policy context and factors influencing policy development. |
|  | Multi-Stakeholder Involvement | None | Considers stakeholders as part of the governance dimension included in the framework in relation to the policy mixes are designed and the policy rationale. However, it is not an actor-centric framework and misses an analysis of actor roles, networks and power dynamics. |
|  | Policy Coherence and Integration | Full | The framework explicitly focuses on coherence across policy instruments and how well they align with broader strategic goals. It facilitates the analysis of consistency and credibility across policy mixes and processes and allows for both horizontal and vertical coherence analysis. |
| ‘Full’ indicates a framework that facilitates a comprehensive food systems analysis of the criterion. ‘Partial’ indicates a framework that includes a prompt for the criterion analysis but less comprehensive in terms of ‘food systems’ analysis. ‘None’ indicates a framework that does not include a prompt for the criterion.*Criteria are based on definitions of integrated food policy^(4)^ and recommendations for integrated food policymaking in the Organisation for Economic Co-operation and Development (OECD )‘Making Better Policies for Food Systems’ report^(5)^. | | | |

**References for supplementary table S1.**

1. Sabatier PA.(1993) *Policy change and learning : an advocacy coalition approach* . Westview Press.

2. Resnick D, Haggblade S, Babu S, *et al.* (2018) The Kaleidoscope Model of policy change: Applications to food security policy in Zambia. *World Dev* 109, 101–20. doi: 10.1016/j.worlddev.2018.04.004.

3. Ouedraogo O, Doudou MH, Drabo KM, *et al.* (2020) Policy overview of the multisectoral nutrition planning process: The progress, challenges, and lessons learned from Burkina Faso. *Int J Health Plann Manage* 35(1), 120–39. doi: 10.1002/hpm.2823.

4. Parsons K.(2019) *Brief 3: Integrated Food Policy - What is it and how can it help connect food systems*. London, UK: Centre for Food Policy City University.

5. OECD (2021), *Making Better Policies for Food Systems.* Paris: OECD Publishing. doi: [10.1787/ddfba4de-en](https://doi.org/10.1787/ddfba4de-en).

6. Clark SG (2002) *The Policy Process: A Practical Guide for Natural Resources Professionals*. Yale University Press.

7. Studlar D & Cairney P (2019) Multilevel governance, public health and the regulation of food: is tobacco control policy a model? *J Public Health Policy* 40(2), 147–65. doi: 10.1057/s41271-019-00165-6.

8. Shiffman J & Smith S (2007) Generation of political priority for global health initiatives: a framework and case study of maternal mortality. *Lancet* 370(9595), 1370–9. doi: 10.1016/S0140-6736(07)61579-7.

9. Pawson R & Tilley N (1997) *Realistic Evaluation*. Sage.

10. Timotijevic L, Brown KA, Lähteenmäki L, *et al.* (2013) EURRECA-A framework for considering evidence in public health nutrition policy development*. Crit Rev Food Sci Nutr* 53(10), 1124–34. doi: 10.1080/10408398.2012.747485..

11. Kingdon, J (1984) *Agendas, Alternatives, and Public Policies*. Harper Collins.

12. Geels FW.(2002) Technological transitions as evolutionary reconfiguration processes: a multi-level perspective and a case-study. *Research Policy*, 31(8), 1257–1274. doi:10.1016/S0048-7333(02)00062-8.

13. Court J & Young J (2006) Bridging Research and Policy in International Development: An Analytical and Practical Framework. *Dev Pract* 16(1):85–90.

14. Feindt PH, Schwindenhammer S, Tosun J (2021) Politicization, Depoliticization and Policy Change: A Comparative Theoretical Perspective on Agri-food Policy. *J Comp Policy Anal Res Pract* 23(5–6), 509–25. doi: 10.1080/13876988.2020.1785875.

15. Hebinck A & Page D (2017) Processes of Participation in the Development of Urban Food Strategies: A Comparative Assessment of Exeter and Eindhoven. *Sustainability* 9(6), 931. doi: 10.3390/su9060931..

16. Gillespie S, Haddad L, Mannar V, *et al.* (2013) The politics of reducing malnutrition: building commitment and accelerating progress. *Lancet* 382(9891),552–69. doi: 10.1016/S0140-6736(13)60842-9.

17. Kugelberg S, Bartolini F, Kanter DR, *et al.* (2021) Implications of a food system approach for policy agenda-setting design. *Glob Food Secur* 28, 100451. doi: 10.1016/j.gfs.2020.100451.

18. Cervantes G, Thow AM, Gómez-Oliver L, *et al.* (2021) What Opportunities Exist for Making the Food Supply Nutrition Friendly? A Policy Space Analysis in Mexico. *Int J Health Policy Manag* 11(11), 2451–63. doi: 10.34172/ijhpm.2021.164

19. Ham C & Hill M.(1993) *The Policy Process in the Modern Capitalist State*. London, UK: Wheatsheaf Books.

20. Walt G & Gilson L (1994) Reforming the health sector in developing countries: the central role of policy analysis. *Health Policy Plan* 9(4), 353–70. doi: 10.1093/heapol/9.4.353

21. Minotti B, Cimini A, D’Amico G, et al. (2022) Food Policy Processes in the City of Rome: A Perspective on Policy Integration and Governance Innovation. Front Sustain Food Syst 5. doi: 10.3389/fsufs.2021.786799

22. Galli F, Prospero P, Favilli E, *et al.* (2020) How can policy processes remove barriers to sustainable food systems in Europe? Contributing to a policy framework for agri-food transitions. *Food Policy* 96, 101871. doi: 10.1016/j.foodpol.2020.101871.

23. Bacchi C. (2009) *Analysing Policy: What’s the Problem Represented To Be?* Australia: Pearson Education.

24. Biesbroek R & Candel JJL (2020) Mechanisms for policy (dis)integration: explaining food policy and climate change adaptation policy in the Netherlands. *Policy Sci* 53(1), 61–84. doi: 10.1007/s11077-019-09354-2.

25. Rogge K & Reichardt K (2016) Policy mixes for sustainability transitions: An extended concept and framework for analysis. *Res Policy* 45(8),1620–35. doi: 10.1016/j.respol.2016.04.004.
